# Fluorescent carbon dot–molecular salt hydrogels†
†Electronic supplementary information (ESI) available: Fluorescent emission spectra of CD systems and tables of gelation experiments and single crystal structure determination in CIF format. CCDC 1402495. For ESI and crystallographic data in CIF or other electronic format see DOI: 10.1039/c5sc01859e


**DOI:** 10.1039/c5sc01859e

**Published:** 2015-07-29

**Authors:** Angelina Cayuela, Stuart R. Kennedy, M. Laura Soriano, Christopher D. Jones, Miguel Valcárcel, Jonathan W. Steed

**Affiliations:** a Department of Analytical Chemistry , Marie Curie Building , Campus de Rabanales , University of Córdoba , E-14071 Córdoba , Spain . Email: qa1vacam@uco.es ; Tel: +34 957 218616; b Department of Chemistry , University of Durham , South Road , DH1 3LE , UK . Email: jon.steed@durham.ac.uk ; Fax: +44 (0)191 384 4737 ; Tel: +44 (0)191 334 2085

## Abstract

The incorporation of functionalised carbon nanodots within a novel low molecular weight salt hydrogel derived from 5-aminosalicylic acid is reported. The carbon dots result in markedly enhanced gelation properties, while inclusion within the hydrophobic gel results in a dramatic fluorescence enhancement for the carbon nanomaterials. The resulting hybrid CD gels exhibit a useful sensor response for heavy metal ions, particularly Pb^2+^.

## Abstract 2

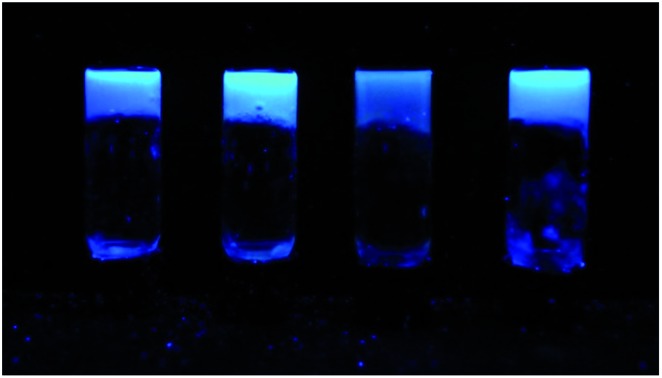
We report the incorporation of functionalised carbon nanodots within a low molecular weight salt hydrogel enhancing the gelation and fluorescence properties of both the gel and carbon nanomaterial.

## Introduction

Impressive progress has been made in photoluminescent (PL) carbon-based dots (CDs) since their discovery in 2004,^1^ in many applications such as bioimaging, drug delivery and analytical sensing. Particular applications include engineering bright nanoprobes towards pollutants and metal ions^2^–^7^ because of their stability, high water solubility, biocompatibility low toxicity, easy surface modification and excellent PL properties. To date CDs have been used in aqueous solution, however incorporating them into a gel medium offers particular promise in stabilising and immobilising the nanoparticles, and may result in interesting effects on the properties of the gel–nanomaterial hybrid itself. Moreover, shielding the CDs within a more hydrophobic environment may result in considerable enhancement of the CD fluorescence. Recently, a few researchers have incorporated carbon nanomaterials in ionic liquids or into agarose hydrogels to modify their electrochemical properties,^8^ as part of a sensing system for heavy metal ions^9^ or as a vehicle for drug delivery.^10^

Gels are flexible, soft materials that behave as solids on the analytical timescale, but are composed predominantly of a fluid phase.^11^,^12^ There have been a number of interesting recent reports concerning the incorporation of nanomaterials into gel phase media.^13^–^19^ In particular, introduction of carbon nanomaterials such as carbon nanotubes and graphene has been shown to result in gel stabilization and modification of the carbon nanomaterial properties.^13^ There has been considerable recent interest in small-molecule supramolecular gels composed of self-assembled fibrillar networks (SAFINs) derived from relatively well-defined interactions between low molecular weight gelators (LMWG).^11^,^20^–^29^ These small-molecule SAFINs offer the opportunity to design desirable gel properties. Gelator preparation is generally straightforward allowing considerable synthetic versatility and variability, and the gels often exhibit readily reversible gelation behaviour.^12^ The range of structure types of LMWG is very broad, comprising amides, nucleobases, fatty acids and dendrimers, for example.^12^ Within this context, bis(urea) derivatives^30^–^34^ are particularly versatile because the gel-forming tendency of the urea groups is tolerant of variations in the spacer between them and the peripheral substituents, allowing the design of tailored gelators for targeted applications such as separations,^35^ drug delivery,^36^–^38^ polymer templating^39^,^40^ and as media to control crystal growth.^41^–^44^

In this work we report the preparation, properties and ion sensing applications of novel carbon dot–hydrogel hybrid nanomaterials using LMWGs designed to provide a hydrophobic environment suitable for fluorescent ion sensing within an aqueous medium. While polymer gels have been used in conjunction with CDs,^9^,^18^,^19^ to the best of our knowledge this work represents the first incorporation of CDs within an LMWG-based gel medium.

## Results and discussion

### Synthesis and characterization of bis(urea) gels

The bis(urea) gelators of type **1** are based on a bolaamphiphile design^45^ that incorporates a hydrophobic diphenylmethane-derived central spacer (which we have found commonly results in robust gelation characteristics in bis(urea) systems^46^) in conjunction with ionisable salicylic acid based peripheral substituents capable of imparting water solubility. Related urea and amide carboxylic acid systems have been studied previously.^47^–^54^ The bis(2-ureido-2-propyl)-*m*-phenylene analogues of type **2** represent comparator examples that are expected to be poor gelators as a result of the steric hindrance around the urea carbonyl group. As a result compounds with this spacer group are frequently more crystalline than most bis(ureas). In previous work we have found they act as useful model compounds allowing structural insight into the system because they are easier to isolate in a form suitable for single crystal X-ray diffraction studies.

**Fig. 1:**
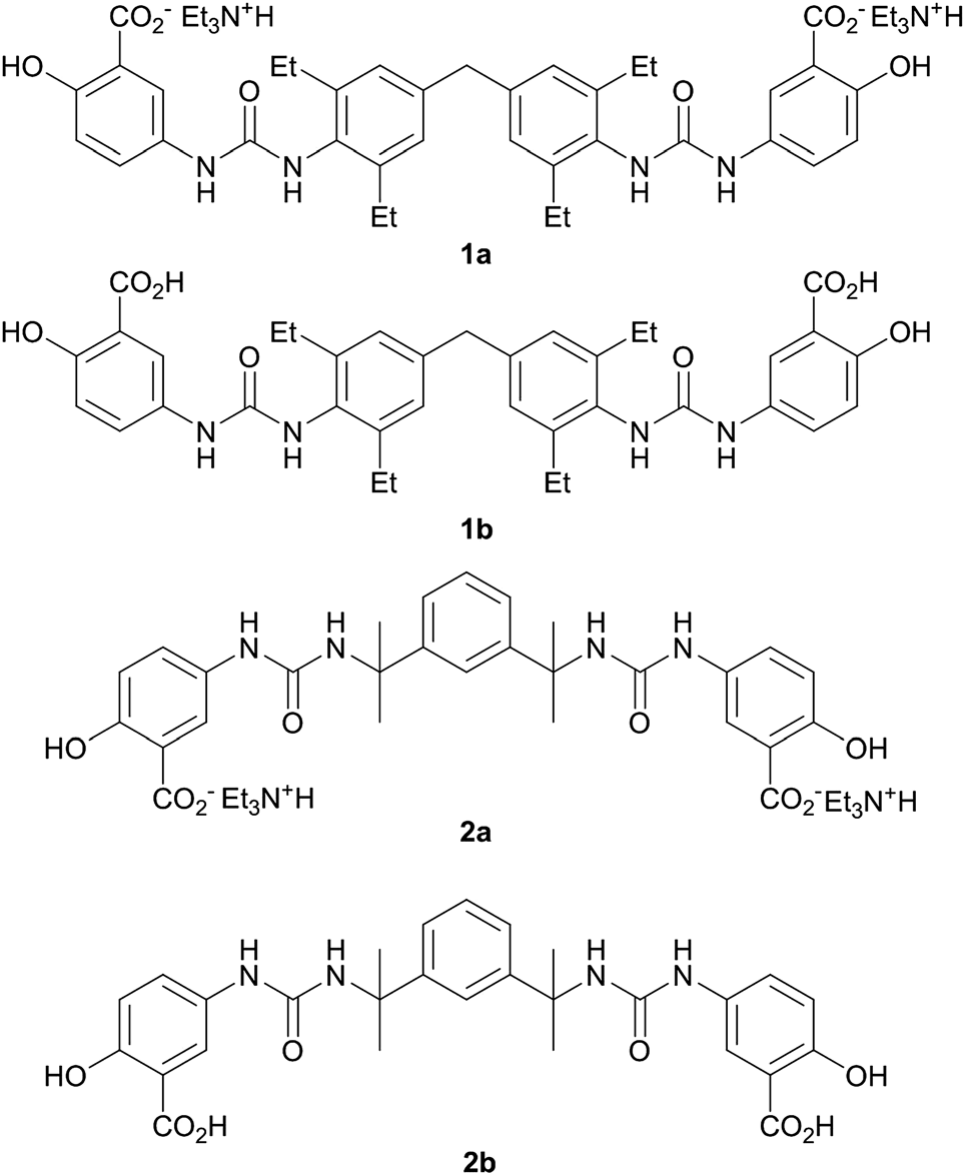
The triethylammonium salt gelators **1a** and **2a**, and the analogous free acids **1b** and **2b**.

The synthesis of the salt gelators **1a** and **2a** was carried out in a one-step reaction between the desired isocyanate and 5-aminosalicylic acid in the presence of triethylamine base. The presence of the triethylammonium cations and the loss of the acid proton in salts **1a** and **2a** was clearly evident in the ^1^H NMR spectra and analytical data of the materials, and was confirmed by X-ray crystallography in the case of **2a** (*vide infra*), Fig. 1. The analogous free acid **1b** was prepared by sonicating **1a** in 1 M hydrochloric acid for 30 minutes. Compound **2b** was prepared by dissolving **2a** in water followed by the addition of 1 M hydrochloric acid upon which the compound precipitated.

**Fig. 2:**
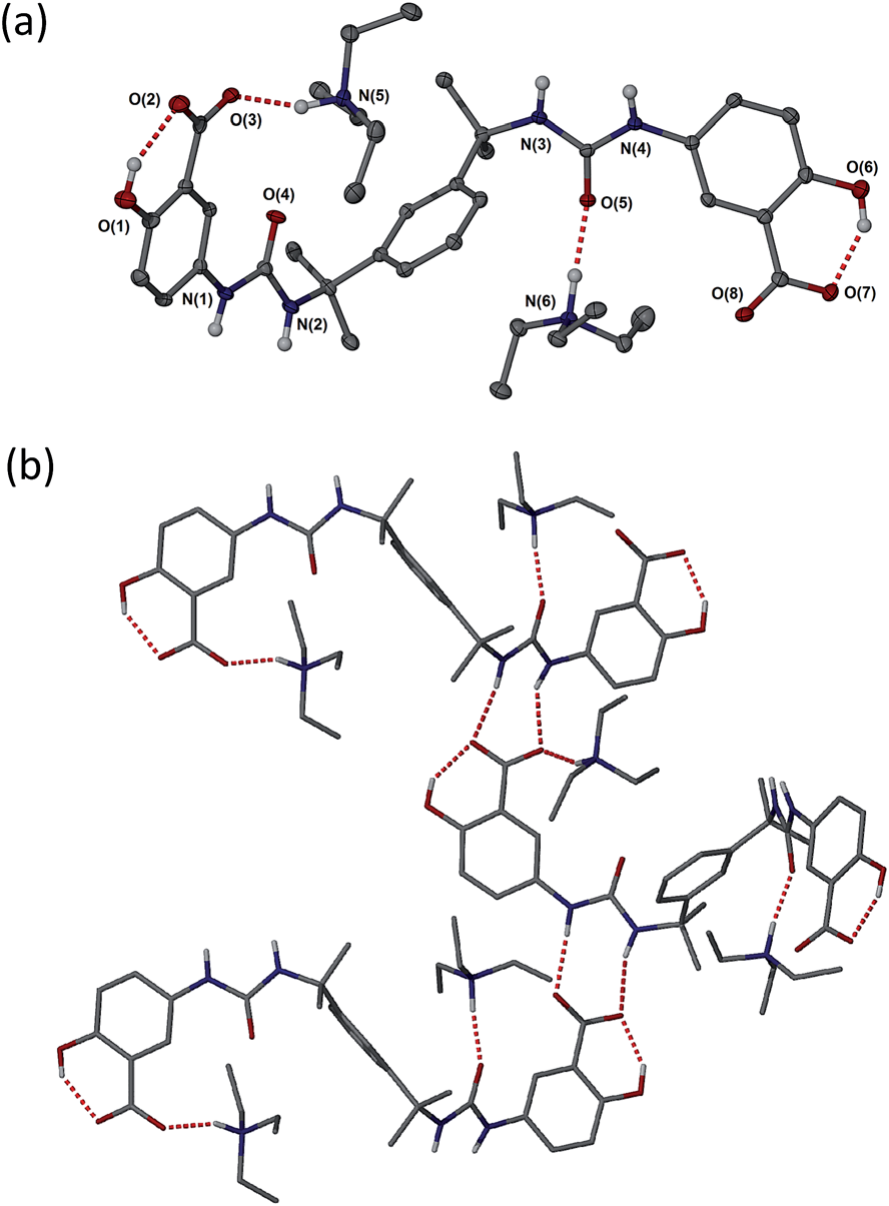
(a) Molecular structure of **2a**·propan-1-ol (disordered propan-1-ol and CH hydrogen atoms omitted). (b) Hydrogen bonding in the structure of **2a**·propan-1-ol. Selected hydrogen bonding distances (Å): O1···O2 2.577(5), N1···O8 2.811(6), N4···O3 2.832(5), N5···O3 2.700(6), O6···O7 2.505(5) and N6···O5 2.775(5).

Compounds of type **1** and **2** as both free acids and triethylammonium salts were screened for gelation and crystallisation in a wide range of solvents. All gelation experiments were carried out at 1 wt% using 5 mg of **1a** in 0.5 mL of solvent. Samples were sonicated for 30 seconds followed by careful heating in a sealed vial until the solid dissolved. Gelation was observed on cooling to ambient temperature.

**Fig. 3:**
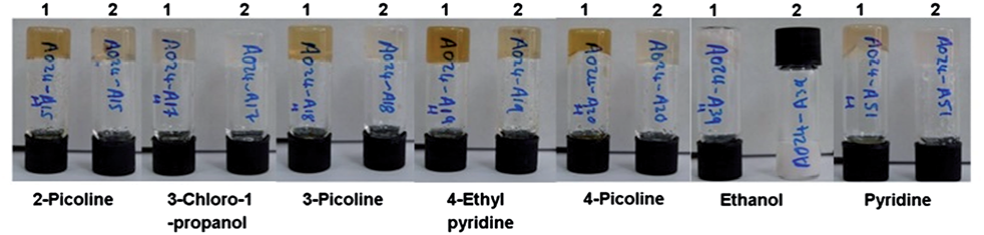
Comparison between gels of **1a** formed by a combination of ultrasound and heat treatment (labelled 1) and room temperature sonogels (2) at 1 wt%.

Compound **1** proved to be a versatile gelator in both salt (**1a**) and neutral (**1b**) forms. Salt **1a** forms gels in a variety of solvents outlined in Table S1 (ESI†). Gels formed upon heating and subsequent cooling in a total of 26 out of 50 solvents studied and partial gels were also observed in 4 solvents. The compound gels a range of polar solvents with both hydrogen bond donor (*e.g.* alcohols) and acceptor character (*e.g.* pyridine derivatives). The neutral acid form **1b** gels in 12 of the 50 solvents studied and partial gels were also observed in 10 solvents outlined in Table S2 (ESI†). Interestingly, room temperature gelation of **1a** on sonication was also observed in a number of instances (Table S3†).^55^,^56^ Comparison between room temperature sonogels and gels produced by a combination of ultrasound and thermal methods revealed that the thermally treated materials are more robust and longer lived, although some minor degradation was evident in the colouration of some gels (Tables S1 and S3,† Fig. 3). Some of the gels formed by the sonication-only process appeared to collapse more readily and in the case of ethanol, only persist for around one hour.

While compound **1b** gels only organic solvents, the behaviour of salt **1a** in water is particularly intriguing with no gelation observed for several days after dissolution at 1 wt%. However from 72 hours onwards partial gels began to form and after 10 days robust, transparent self-supporting gels form implying very slow fibre assembly within the competitive aqueous medium (ESI, Fig. S5†). Increasing the concentration to 2 wt% results in gelation in less than one day. Gelation is also markedly accelerated by lowering the pH (gelation at 1 wt% is rapid in the presence of 1 μL of concentrated sulphuric acid). Subsequent addition of base in the form of NEt_3_ results in collapse of the gel.

As anticipated, compound **2** with its bulky spacer did not form gels in any solvents (Tables S4 and S5†), an outcome attributed to steric hindrance of the urea carbonyl group preventing urea tape formation (in comparison the aromatic groups of **1** may rotate to be orthogonal to the urea plane). Crystals of **2a** suitable for single crystal X-ray structure determination were obtained by slow evaporation of a solution in propan-1-ol. While not from a gel, the crystal structure serves to provide insight into the kinds of interactions possible between gelators bearing these functional groups, the triethyl ammonium counter cation and the solvent. The crystals proved to be a 1 : 1 solvate with two crystallographically unique HNEt_3_^+^ cations hydrogen bonded to one carboxylate group and one urea carbonyl group of a single unique bis(urea) molecule in the presence of a highly disordered molecule of propan-1-ol. The solvent molecule hydrogen bonds to a phenolic hydroxyl group. Typically gels of bis-ureas arise from the formation of α-tape hydrogen bonding interactions between the urea groups in neighbouring molecules based on a six-membered hydrogen bonded ring.^34^,^57^,^58^ In the present structure however, the Et_3_NH^+^ cations effectively inhibit urea tape formation by hydrogen bonding to one of the urea groups and sterically blocking the other. Instead, the strong hydrogen bond acceptor nature of the carboxylate groups results in the formation of an alternative, twisted non-planar 8-membered hydrogen-bonded ring between the urea functionalities and the carboxylate groups (Fig. 2b). The phenolic OH groups are both involved in intramolecular hydrogen to the carboxylate acceptors.

### Carbon dot hybrid gels

Despite the slow assembly of hydrogels of **1a** the final gels proved to be robust and transparent and hence were investigated as host media for fluorescent carbon dots. Four different types of carbon dot were investigated. High luminescence CDs (c-CDs) were prepared from microcrystalline cellulose (MCC) whereas low luminescence CDs were obtained from multi-walled carbon nanotubes (MWCNTs). The nanotube-derived CDs were passivated with acetone (p-CDs) and in some cases subsequently functionalised to give thiol rich (t-CDs) or amine rich surfaces (a-CDs) to improve their PL properties and ability to interact with the gel and with potential analytes. CD-gel hybrids were prepared by sonicating 0.5 to 4 wt% of **1a** in aqueous solutions of the carbon dots ranging from 1–60 mg mL^–1^. The critical gelation concentration proved to be 1 wt% with solutions of 0.5 wt% not forming self-supporting gels.

Remarkably, unlike the hydrogels which form over a period of 10 days at 1 wt%, gelation of all of the CD–gel hybrids occurred in a matter of minutes. This fortuitous result renders the hybrid gels much more convenient to prepare and study than the pure hydrogels and hence is of marked benefit in a practical sensing context. While the mechanism for gelation is not known the CDs may well act as a hydrophobic nucleation and cross-linking node for SAFIN assembly interacting with the fibres by hydrophobic and π–π stacking interactions. The effect is not related to the increased gelation propensity on acidification since the CD solutions are slightly basic, pH 8–9. Gels containing high concentrations of CDs proved to be opaque because of the very intense absorption of the CDs, however dilution to 1 wt% solutions containing 1 mg mL^–1^ CDs gave transparent gels suitable for fluorescence study, although at this low CD concentration gelation was less readily reproducible than at higher concentrations. We postulate that hydrogen bonding to the solubilising Et_3_NH^+^ cations may interfere with the gel assembly process and accordingly we investigated counter cation metathesis by adding a water soluble ionic liquid 1-butyl-3-methylimidazolium tetrafluoroborate (BMIM-BF_4_). The BMIM cation does not possess hydrogen bond donor functionality and hence should not interfere with gelation. Pure ionic liquids such as BMIM-BF_4_ are of interest as ionogels for hosting magnetic nanoparticles, for example, and silicon-functionalised carbon dots have previously been incorporated into ionic liquid based ionogels.^59^–^61^ Compound **1a** does not gel pure BMIM-BF_4_; however, addition of 1–40 wt% of the ionic liquid to the gel–CD mixture gave robust, reproducible gels for all types of CDs. The metathesis with BMIM cations proved highly effective with the resulting hybrid **1a**–CD–BMIM hydrogels forming rapidly at an optimum concentration of 2 wt% BMIM-BF_4_ to give robust, transparent hybrid nanomaterials materials suitable for fluorescence study.

**Fig. 4:**
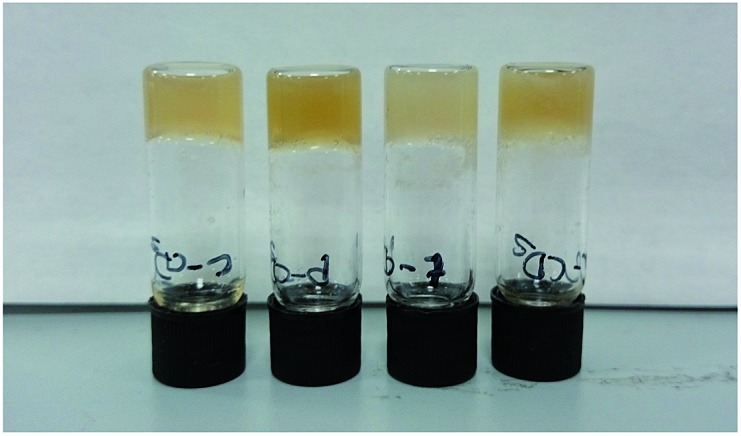
Optimised ionic-liquid CD–Gel hybrid materials of composition 1 wt% **1a** in 500 μL of CD solution at 1 mg mL^–1^ containing 2% BMIM-BF_4_ (left-to-right c-CD, p-CD, t-CD and a-CD).

**Fig. 5:**
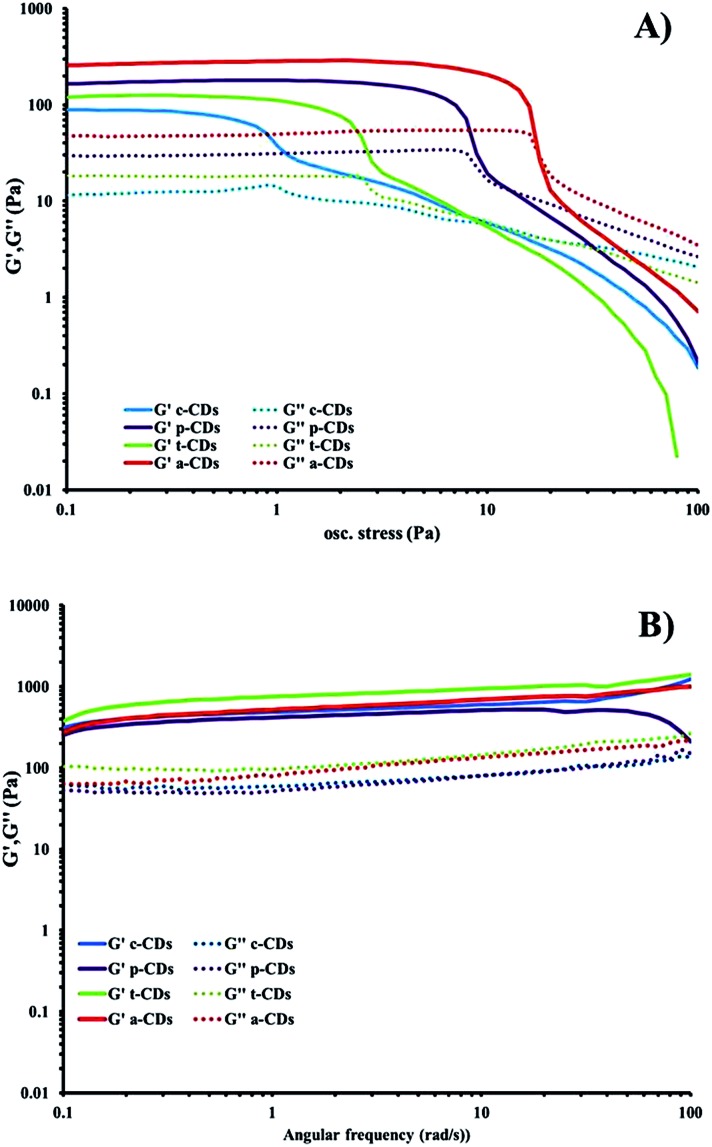
(a) Stress sweep and (b) frequency-sweep rheology of dilute ionic-liquid CD–gel hybrid materials of composition 1 wt% **1a** in 500 μL of CD solution at 1 mg mL^–1^ containing 2% BMIM-BF_4_.

The optimised nanomaterials of composition 1 wt% **1a** in 500 μL of CD solution at 1 mg mL^–1^ containing 2% BMIM-BF_4_ are shown in Fig. 4. The hybrid gels were characterised by frequency and stress sweep rheometry and by SEM. The rheological properties of the four different CD hybrid materials were similar to one another with all exhibiting an elastic modulus *G*′ greater than the viscous modulus *G*′′, with *G*′ invariant with frequency, Fig. 5. At the concentration used the gels are relatively weak with *G*′ values of 100–300 Pa and yield stress around 1–10 Pa. The gels containing the amine-functionalised CDs proved generally to be considerably more robust that the unfunctionalised c-CDs which may indicate a specific hydrogen bonding interaction between the amine groups and the carboxylate functionalities of the gelators. The SEM images of the dried gels showed a fibrous structure consistent with similar SAFIN-based hydrogels^62^ with small fibres of average diameter of less than 30 nm consistent with the transparent appearance of the gels. In comparison the hydrogels before introduction of CDs give slightly larger, more tape-like fibres (ESI Fig. S4†).

### CD-gel fluorescence

**Fig. 6:**
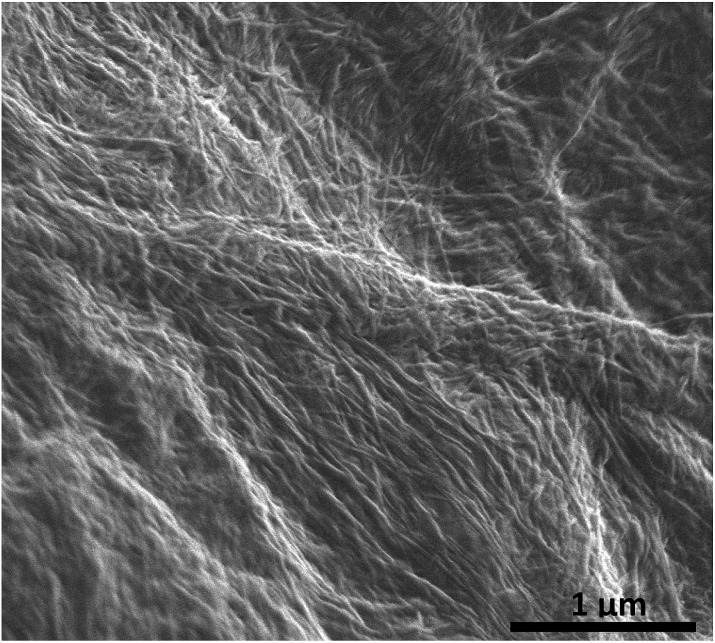
SEM micrograph of the chromium coated dried (xerogel) ionic-liquid a-CD–gel hybrid materials based on **1a**. The carbon dots of average radius 3 nm are too small to be observed.

All the CDs in aqueous solution were fluorescent with a full width at half maximum (FWHM) about 100 nm which indicates their homogeneity. The bottom-up synthesised c-CDs proved to be the most fluorescent and are known to possess a higher quantum yield.^63^ The maximum excitation wavelength was 370 nm, shifting to 365 for the p-CDs, t-CDs and a-CDs. Fig. S1† shows the fluorescent spectra of all the CD solutions. Consistent with previous reports,^64^–^66^ the functionalised CDs were more fluorescent than the passivated p-CDs (Fig. 6).

**Fig. 7:**
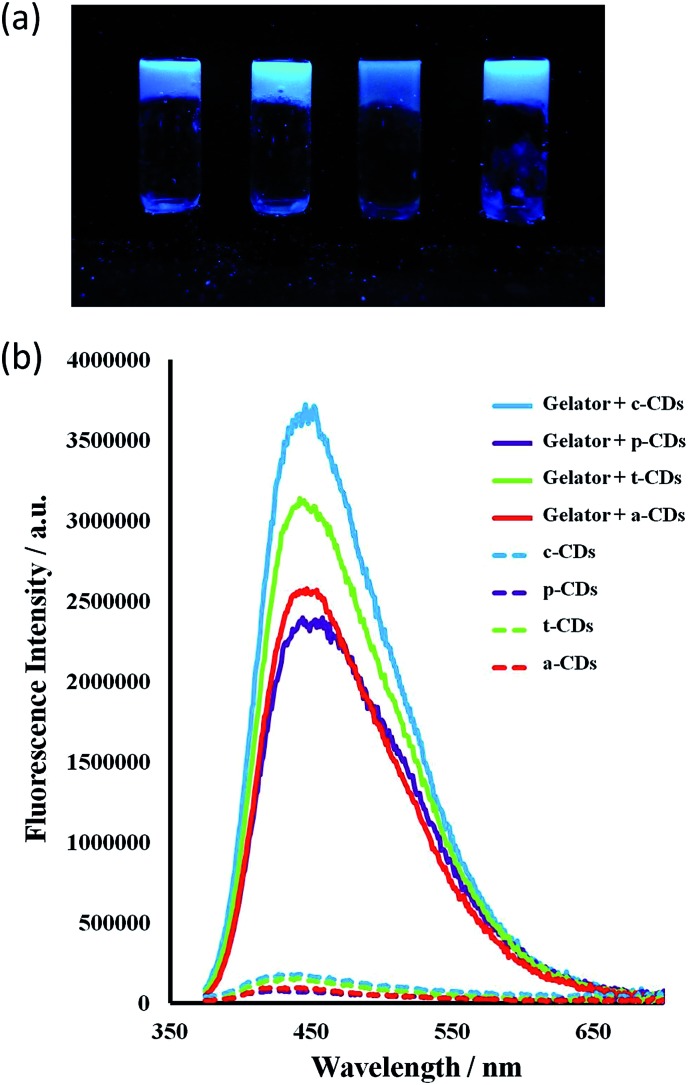
(a) a-, c-, p- and t-CD gels 1 wt% under 365 nm UV irradiation. (b) Fluorescence emission spectra of aqueous CD solutions (1 mg mL^–1^, *λ*_ex_ = 365 nm for c-CDs and 370 nm for the others) and for 1 wt% CD-gels with **1a** at the same CD concentration. Slits at 2 nm.

Incorporation of the CDs into the gels resulted in a striking enhancement of the CD emission intensity by at least an order of magnitude for all types of CDs studied. It is well-known that the PL intensity varies significantly with the size and core structure due to the synthetic route and precursor used, the surface functionalities of CDs, as well as with the environment. As the nanodots in this case are all of similar size, the strong emission enhancement of CDs comes from the interaction of the CDs with the gelator instead of a quantum size effect. Fluorescence spectra for 1 wt% gels of 1 mg mL^–1^ CD solutions in the absence of the ionic liquid are shown in Fig. 7. The ionic liquid itself is also fluorescent, complicating the spectra, however the marked emission enhancement is also evident in the ionic liquid CD–gel hybrid materials, Fig. S2 (ESI†). The hydrophobic nature of the gels is likely to significantly reduce solvent quenching and the fact that the emission enhancement is so marked suggests significant interaction between the gel fibres and the CDs in a synergistic way, consistent with the marked effect the CDs have on the gelation properties.

**Fig. 8:**
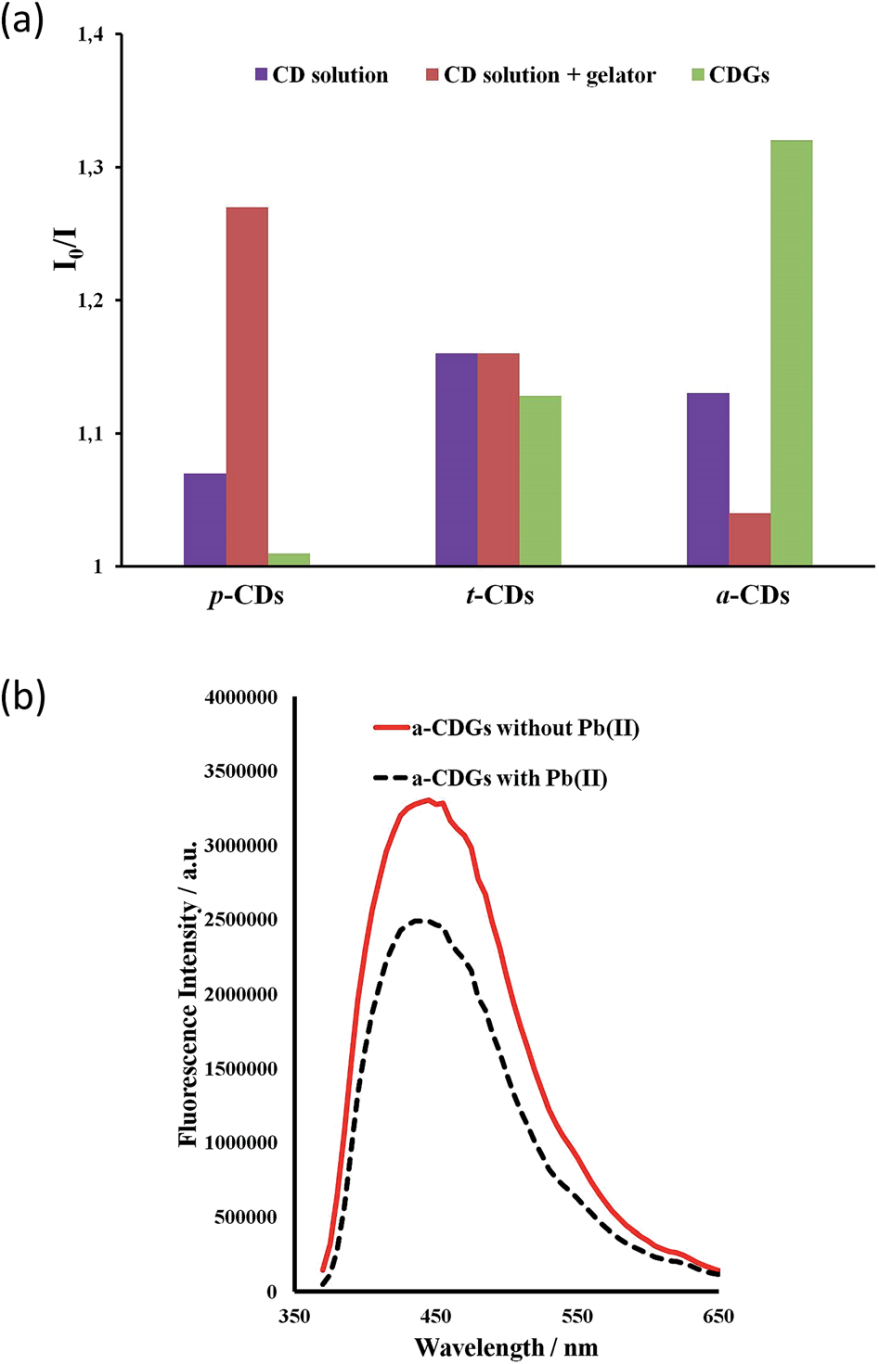
(a) Photoluminescent response to 10 μg mL^–1^ of Pb^2+^ to CD solutions, CD-containing hydrogels (CDGs) and CD solutions in the presence of dissolved gelator (1 mg mL^–1^, *λ*_ex_ = 370 nm) and for 1 wt% CD–gels with **1a** at the same CD concentration. (b) Fluorescence emission spectra for a-CD gel samples with and without Pb^2+^ (10 μg mL^–1^).

The high intensity fluorescence of these novel CD gel hybrids and the possibility of tuning the properties of the materials by modification of the gelator, imidazolium cation and the size and surface functionality of the CDs offers interesting possibilities for their application as luminescent sensors, *e.g.* as environmental nanoprobes. CDs have been shown to be an excellent sensing platform for heavy metals,^63^,^64^ and the interaction of metal ions with the CD surface functionality may well be significantly influenced by the inclusion of the CDs within a LMWG hydrogel medium. In order to establish the feasibility of heavy metal sensing using hybrid CD gels the effect of added lead(ii) and mercury(ii) on the PL of the new hybrid gel system was investigated. The fluorescence of aqueous p-CD, t-CD and a-CDs solutions is moderately quenched by 10 μg mL^–1^ of Hg^2+^ and Pb^2+^ (*I*_0_/*I* = 1.1–1.25) while the luminescence of c-CDs is not affected. In the hybrid CD gel system, in addition to the marked increase in overall PL intensity, the response to Hg^2+^ and Pb^2+^ changes differentially. The response to Pb^2+^ of the gels containing p-CDs and t-CDs is markedly attenuated such that the luminescence is essentially unaffected, whereas the system containing the a-CDs exhibits a marked enhancement in its Pb^2+^ response in the gel phase (*I*_0_/*I* increases from 1.13 to 1.32), Fig. 8. In contrast the a-CD gel exhibits essentially no response to Hg^2+^. This differential behaviour suggests that the CD gels may provide a basis for discriminating closely related heavy metal ions in the environment. As a control the metal ion response was also examined in CD solution in the presence of the gelator without gel formation. The presence of the gelator in solution had no effect on the t-CD response, attenuated the response of the a-CD system and enhanced the response of the p-CDs. The significant and differential difference in sensor behaviour in the gel state is an interesting phenomenon. To further probe selectivity the response of a-CD-containing hydrogels (a-CDGs) as sensing probe towards a range of metal ions, namely Pb^2+^, Hg^2+^, Co^2+^, Fe^3+^ and Mg^2+^ was examined (ESI, Fig. S3†). The system proved to be clearly Pb^2+^ selective in the hydrogel state. The a-CDGs thus represent a new format of selective sensor for the detection of lead(ii). In future work we will systematically explore the fluorescent response of these novel hybrid nanomaterials to a broader range of analytes. A further interesting application platform could also involve the use of freeze dried hybrid CD hydrogels coated with TiO_2_ allowing them to float on water as reporter medium.^67^

## Conclusions

Inclusion of luminescent carbon dots within a hydrophobic low molecular weight hydrogel results in a dramatic enhancement of their luminescence intensity as well as an unexpected strengthening of the gels and marked increase in their rate of self-assembly. In the gel phase the CDs exhibit a differential photoluminescent response to the presence of heavy metal ions. The novel hybrid CD–gel nanostructures reported herein offer interesting potential as highly tunable luminescent media with possible applications in environmental and biomedical heavy metal ion sensing. The potential biocompatibility of these nanostructures which are derived from non-toxic precursors may open a window for their use in medical applications such as diagnosis, drug activation or slow release.

## Experimental

### Reagents and instrumental

Sulphuric acid (95–98%), nitric acid (69%), ethanol (99%) and chloroform were purchased from PANREAC, S.A.U. (Barcelona, Spain). *N*,*N*′-Diisopropylcarbodiimide (DIC, 98%), *N*-hydroxysuccinimide (NHS, 97%), triethylamine (TEA, 99%), cysteamine hydrochloride (98%), *N*-Boc-ethylenediamine (Boc-EDA, 98%), cellulose microcrystalline (50 μm particle size), acetone (99.5%), 1-butyl-3-methylimidazolium tetrafluoroborate (BMIM-BF_4_, 97%), dimethyl sulfoxide (DMSO, 99.9%), sodium carbonate (99.95%). 5-Aminosalicylic acid and the diisocyanates purchased from Sigma–Aldrich, while the multi walled carbon nanotubes were purchased from Bayer (Germany). NMR spectra were measured on a Bruker Avance-400.

### Synthesis

#### Compound **1a**

5-Aminosalycilic acid (0.5 g, 3.27 mmol) was added to a solution of 4,4′-methylenebis(2,6-diethylaniline) (0.59 g, 1.63 mmol) in chloroform : ethanol (15 mL : 1.5 mL). Triethylamine (0.78 mL, 5.59 mmol) to give a solution that was heated under reflux for 3 h. The resulting suspension was then filtered and washed with chloroform (100 mL) to give **1a** as an off-white solid (0.95 g, 1.09 mmol, 67%). ^1^H NMR (400 MHz, DMSO-*d*_6_) *δ* 8.57 (s, 2H, OH[combining low line]), 7.68 (s, 2H, NH[combining low line]), 7.62 (s, 2H, NH[combining low line]) 7.31 (d, ^3^*J* = 8.7, Hz, 2H, ArH[combining low line]), 6.98 (2s, 6H, ArH[combining low line]), 6.58 (d, ^3^*J* = 8.6 Hz, 2H, ArH[combining low line]), 3.85 (s, 2H, CH[combining low line]_2_), 3.07 (q, *J* = 7.3 Hz, 12H, NCH[combining low line]_2_CH_3_), 2.55 (q, ^3^*J* = 7.7, Hz, 8H, ArCH[combining low line]_2_CH_3_), 1.17 (t, ^3^*J* = 7.1 Hz, 18H, NCH_2_CH[combining low line]_3_), 1.11 (t, ^3^*J* = 7.5 Hz, 12H, ArCH_2_CH[combining low line]_3_); ^13^C{^1^H} NMR (400 MHz, DMSO-*d*_6_) *δ* 172.09 (C[combining low line]O_2_H), 156.28 (C[combining low line]=O), 142.20 (ArC[combining low line]), 140.02 (ArC[combining low line]), 132.64 (ArC[combining low line]), 132.19 (ArC[combining low line]), 126.62 (ArC[combining low line]), 117.43 (ArC[combining low line]), 112.54 (ArC[combining low line]), 41.20 (C[combining low line]H_2_), 24.67 (C[combining low line]H_2_), 15.11 (C[combining low line]H_3_); ESI (*m*/*z*): 770 [M – NEt_3_ + H]^+^; analysis calc. for C_49_H_70_O_8_N_6_: C 67.56, H 8.1, N 9.65%; found: C 66.73, H 7.88, N 9.18%.

#### Compound **1b**

Compound **1a** was sonicated in 1 M aqueous HCl (250 mL) for 30 minutes, filtered and washed with chloroform to give **1b** as an off white solid which was isolated by filtration and air dried (0.6 g, 0.89 mmol, 54.8%). ^1^H NMR (400 MHz, DMSO-*d*_6_) *δ* 10.79 (bs, 2H, CO_2_H[combining low line]), 8.87 (s, 2H, OH[combining low line]), 7.95 (s, 2H, NH[combining low line]), 7.64 (s, 2H, NH[combining low line]), 7.49 (d,^3^*J* = 8.9, Hz, 2H, ArH[combining low line]), 6.98 (s, 6H, ArH[combining low line]), 6.87 (d, ^3^*J* = 8.9 Hz, 2H, ArH), 3.85 (s, 2H, CH[combining low line]_2_), 2.53 (q, ^3^*J* = 7.5 Hz, 8H, ArCH[combining low line]_2_CH_3_), 1.10 (t, ^3^*J* = 7.5 Hz, 12H, ArCH_2_CH[combining low line]_3_); ^13^C{^1^H} NMR (400 MHz, DMSO-*d*_6_) *δ* 172.30 (C[combining low line]O_2_H), 157.40 (C[combining low line]=O), 154.91 (ArC[combining low line]), 142.42 (ArC[combining low line]), 139.78 (ArC[combining low line]), 132.63 (ArC[combining low line]), 130.15 (ArC[combining low line]), 126.66 (ArC[combining low line]), 119.91 (ArC[combining low line]), 115.69 (ArC[combining low line]), 45.93 (C[combining low line]H_2_), 24.99 (C[combining low line]H_2_), 15.17 (C[combining low line]H_3_), 9.10 (C[combining low line]H_3_); ESI (*m*/*z*): 667 [M – H]^–^; analysis calc. for C_37_H_40_O_8_N_4_: C 66.45, H 6.03, N 8.38%; found: C 63.54, H 5.67, N 7.82%. The difference can be accounted for by calculating the formula with a 0.3 molar ratio of CHCl_3_.

#### Compound **2a**

5-Aminosalicylic acid (0.5 g, 3.27 mmol) was added to a solution of 1,3-bis(1-isocyanato-1-methylethyl)benzene (0.38 mL, 1.65 mmol) in chloroform : ethanol (15 mL : 1.5 mL). Triethylamine (0.78 mL, 5.59 mmol) to give a solution that was heated under reflux for 4 h. The resulting suspension was filtered and washed with chloroform (100 mL) to give **2a** as an off-white solid (1.19 g, 1.59 mmol, 97%). ^1^H NMR (400 MHz, DMSO-*d*_6_) *δ* 8.14 (s, 2H, NH[combining low line]), 7.61 (s, 2H, NH[combining low line]), 7.46 (s, 1H, ArH[combining low line]) 7.26–7.13 (m, 5H, ArH[combining low line]), 6.53 (d, ^3^*J* = 8.6 Hz, 2H, ArH[combining low line]), 6.45 (s, 2H, ArH[combining low line]), 3.07 (q, ^3^*J* = 7.2 Hz, 12H, NCH[combining low line]_2_CH_3_), 1.59 (s, 12H, CCH[combining low line]_3_), 1.17 (t, ^3^*J* = 7.0 Hz, 18H, NCH_2_CH[combining low line]_3_); ^13^C{^1^H} NMR (400 MHz, DMSO-*d*_6_) *δ* 172.64 (C[combining low line]O_2_H), 157.36 (C[combining low line]=O), 157.36 (ArC[combining low line]), 148.70 (ArC[combining low line]), 130.51 (ArC[combining low line]), 123.75 (ArC[combining low line]), 122.93 (ArC[combining low line]), 120.91 (ArC[combining low line]), 119.69 (ArC[combining low line]), 115.79 (ArC[combining low line]), 54.88 (C[combining low line]CH_3_), 45.81 (C[combining low line]H_2_) 30.41 (C[combining low line]H_3_) 9.04 (C[combining low line]H_3_). ESI (*m*/*z*): 652 [M – NEt_3_ + H]^+^; analysis calc. for C_40_H_60_O_8_N_6_: C 63.81, H 8.03, N 11.16%; found: C 54.61, H 6.76, N 9.32%. The difference can be accounted for by calculating the formula with a 1.3 molar ratio of CHCl_3_.

#### Compound **2b**

Compound **2a** was dissolved in H_2_O (250 mL) and 1 M HCl (250 mL) was added slowly where a precipitate formed rapidly. The suspension was filtered and washed with chloroform to give **2b** as an off white solid (0.71 g, 1.29 mmol, 79.0%). ^1^H NMR (400 MHz, DMSO-*d*_6_) *δ* 10.83 (bs, 2H, CO_2_H[combining low line]), 8.36 (s, 2H, NH[combining low line]), 7.87 (s, 2H, NH[combining low line]), 7.44 (s, 1H, ArH[combining low line]), 7.33 (dd, ^3^*J* = 8.9, 2.8 Hz, 2H, ArH), 7.27–7.20 (m, 3H, ArH), 6.81 (d, ^3^*J* = 8.9 Hz, 2H, ArH[combining low line]), 6.46 (s, 2H, ArH[combining low line]), 1.59 (s, 12H, CCH[combining low line]_3_). ^13^C{^1^H} NMR (400 MHz, DMSO-*d*_6_) *δ* 172.30 (C[combining low line]O_2_H) 156.13 (C[combining low line]=O) 154.78 (ArC[combining low line]) 148.45 (ArC[combining low line]) 132.69 (ArC[combining low line]) 127.96 (ArC[combining low line]) 126.77 (ArC[combining low line]) 122.98 (ArC) 121.49 (ArC) 119.09 (ArC[combining low line]) 117.47 (ArC[combining low line]) 112.73 (ArC[combining low line]) 55.01 (C[combining low line]) 30.28 (C[combining low line]H_3_); ESI (*m*/*z*): 549 [M – H]^–^; analysis calc. for C_28_H_30_O_8_N_4_: C 61.08, H 5.49, N 10.18%; found: C 58.42, H 5.4, N 9.78%. The difference can be accounted for by calculating the formula with a 0.25 molar ratio of CHCl_3_.

All gelation experiments were carried out at 1 wt% by dissolving 5 mg of the gelator in 0.5 mL of the desired solvent. The vial was then sealed, sonicated for 30 seconds and heated. Gels formed on standing and cooling to room temperature over a period of minutes to hours. Gel formation was determined by the inversion test.

### Single crystal X-ray diffraction studies

Single crystal data was collected at 120.0(2) K on a Bruker D8Venture diffractometer (PHOTON-100 CMOS detector, IμS-microsource, focusing mirrors, *λ*MoKα, *λ* = 0.71073 Å) and processed using Bruker APEX-II software. The temperature of the samples was maintained by the Cryostream (Oxford Cryosystems) open-flow nitrogen cryostat. The structure was solved by direct method and refined by full-matrix least squares on *F*^2^ for all data using Xseed, OLEX2 and SHELXTL software. All non-disordered non-hydrogen atoms were refined anisotropically, hydrogen atoms were placed in the calculated positions. Site occupation factors of the carbon atoms of disordered propan-1-ol molecule were fixed at 0.50, 0.30 and 0.20, the disordered atoms were refined isotropically.

Crystals of **2a** were prepared by allowing a propan-1-ol solution of the sample to slowly evaporate. Crystal data for **2a**: C_43_H_68_N_6_O_9_, *M* = 813.03, 0.154 × 0.225 × 0.254 mm^3^, monoclinic, space group *P*2_1_ (no. 4), *a* = 8.3147(6), *b* = 19.2037(13), *c* = 14.1567(10) Å, *β* = 102.128(2)°, *V* = 2210.0(3) Å^3^, *Z* = 2, *D*_c_ = 1.222 g cm^–3^, *F*_000_ = 880, MoKα radiation, *λ* = 0.71073 Å, *T* = 120(2) K, 2*θ*_max_ = 46.0°, 18 991 reflections collected, 6058 unique (*R*_int_ = 0.0783). Final GooF = 1.001, *R*_1_ = 0.0570, w*R*_2_ = 0.1421, *R* indices based on 5189 reflections with *I* > 2*σ*(*I*) (refinement on *F*^2^), 540 parameters, 7 restraints. Lp and absorption corrections applied, *μ* = 0.086 mm^–1^. Absolute structure parameter = 0.1(12).

### Carbon dot solutions

Four different types of CDs were synthesised as described previously.^22^–^25^ Briefly, CDs were prepared by acid thermo-hydrolyses using microcrystalline cellulose (MCC) or multi-walled carbon nanotubes (MWCNTs) as carbon precursors. Highly luminescent dots (c-CDs) were obtained using MCC, while less fluorescent CDs were obtained from MWCNTs. Later, the last CDs were passivated with acetone (p-CDs) to boost their PL properties. The p-CDs were then surface functionalised either with cysteamine to give a thiol-rich surface (t-CDs) or *N*-Boc-ethylenediamine to give an amine rich surface (a-CDs). All the CDs synthesised were neutralised and purified by precipitation with ethanol at low temperatures.

### Preparation and optimization of the CD–salt hydrogels

Gels were prepared by dissolving a carefully weighed amount of bis(urea) **1** into an aqueous solution of the relevant CD heating until the gelator dissolved and sonicating the as-prepared mixture three times for few seconds each time. This procedure resulted in gel formation over the course of a few minutes, however at low concentrations of the gelator (≤1 wt%) in the presence of 1 mg mL^–1^ CD solution the gels proved relatively weak. Addition of BMIM-BF_4_ (2 wt%) to metathesise the hydrogen bonding triethyl ammonium cation resulted in stable, robust and transparent gels.

### Fluorescence studies

The differences in the fluorescence intensity and the maximum excitation wavelength between liquid and solid were studied for: c-CD, p-CD, t-CD and a-CD containing gels in comparison with aqueous solutions. Each measurement was carried out three times, *λ*_ex_ = 365 and 370 nm, 2 nm excitation and emission slit widths. Measurements were performed with a PTI QuantaMaster™ Spectrofluorometer.

### Analyses of metal ions

Aliquots of standards (Pb^2+^, Hg^2+^, Co^2+^, Fe^3+^ and Mg^2+^ at 10 μg mL^–1^) were dropped onto the CD solution (before gel formation) and shaken for 1 min. In the case of CD gels as fluorescent probes, formation of the gels was performed as previously described.

### Rheology

Rheological measurements were carried out with an AR2000 rheometer at 10 °C. Frequency sweep experiments were performed at a constant oscillation stress of 1 for the angular frequency of 0.1–100, while the stress sweep was performed for the oscillation stress of 0.1–100.

### Scanning electron microscopy

SEM samples were dried in air at room temperature for 2 days, coated with 3 nm of chromium using a Cressington 328 Ultra High Resolution EM Coating System, and imaged using an FEI Helios NanoLab DualBeam microscope in immersion mode, with typical beam settings of 1.5 kV and 0.17 nA.

## Supplementary Material

Supplementary information

Crystal structure data
