# Correction: Fluorescent carbon dot–molecular salt hydrogels

**DOI:** 10.1039/c5sc90052b

**Published:** 2015-09-07

**Authors:** Angelina Cayuela, Stuart R. Kennedy, M. Laura Soriano, Christopher D. Jones, Miguel Valcárcel, Jonathan W. Steed

**Affiliations:** a Department of Analytical Chemistry , Campus de Rabanales , University of Córdoba , Marie Curie Building , E-14071 Córdoba , Spain . Email: qa1vacam@uco.es ; Tel: +34 957 218616; b Department of Chemistry , University of Durham , South Road , DH1 3LE , UK . Email: jon.steed@durham.ac.uk ; Fax: +44 (0)191 384 4737 ; Tel: +44 (0)191 334 2085

## Abstract

Correction for ‘Fluorescent carbon dot–molecular salt hydrogels’ by Angelina Cayuela *et al.*, *Chem. Sci.*, 2015, DOI: 10.1039/c5sc01859e.



## 

Owing to errors during the production of this article, the editorial office omitted the mandatory EPSRC open access data statement from the article text. The following statement should have been included in the original publication:

“The underlying research data for this paper is available in accordance with EPSRC open data policy from DOI: 10.15128/kk91fm25n”.

The Royal Society of Chemistry apologises for these errors and any consequent inconvenience to authors and readers.

