# Supplementary material for: Fluorescent carbon dot–molecular salt hydrogels
Source: Chem Sci. 2015 Jul 29;6(11):6139–46. doi: 10.1039/c5sc01859e (PMC6055089; doi:10.1039/c5sc01859e)
Supplement: Supplementary file 1 [file SC-006-C5SC01859E-s001.pdf]

Electronic Supplementary Material (ESI) for Chemical Science.
This journal is © The Royal Society of Chemistry 2015

Fluorescent Carbon Dot – Molecular Salt Hydrogels

Angelina Cayuela[a], Stuart R. Kennedy[b], M. Laura Soriano[a], Miguel Valcárcel[*a] and Jonathan W. Steed[*b]

Supplementary information

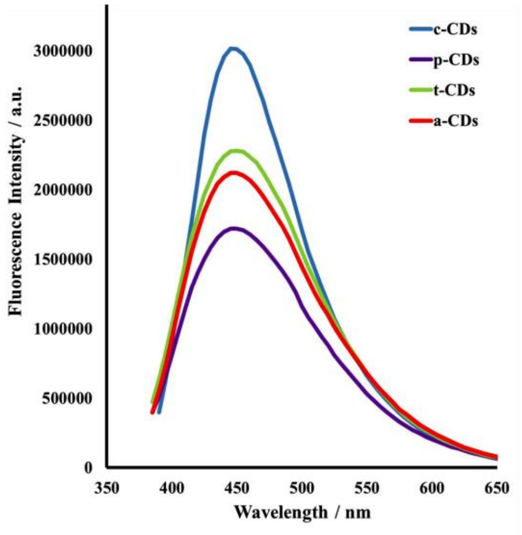

Figure S1. Fluorescence emission spectra of aqueous CD solutions (1 mg mL^{-1}, λ_{ex} = 365 nm for c-CD and λ_{ex} = 370 nm for the others).

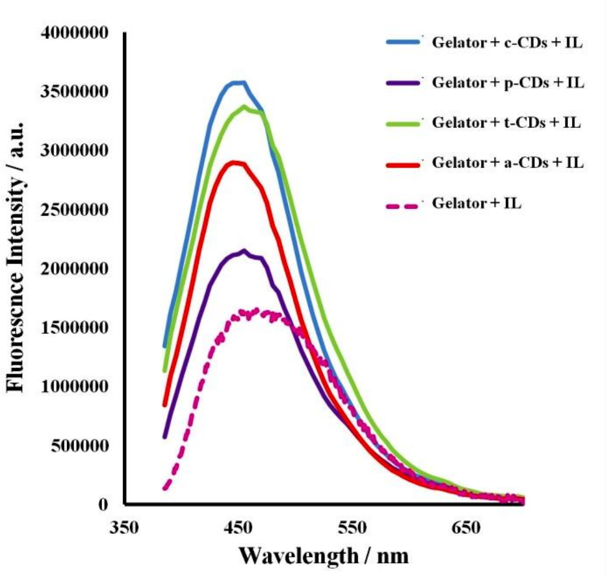

Figure S2. Fluorescence emission spectra of aqueous CD solutions (1 mg mL^{-1}, λ_{ex} = 365 nm for c-CD and λ_{ex} = 370 nm for the others) containing 1 w.t. % CD-gels with **1a** at the same CD concentration and 2% 1-butyl-3-methylimidazolium tetrafluoroborate ($BMIM-BF_4$).

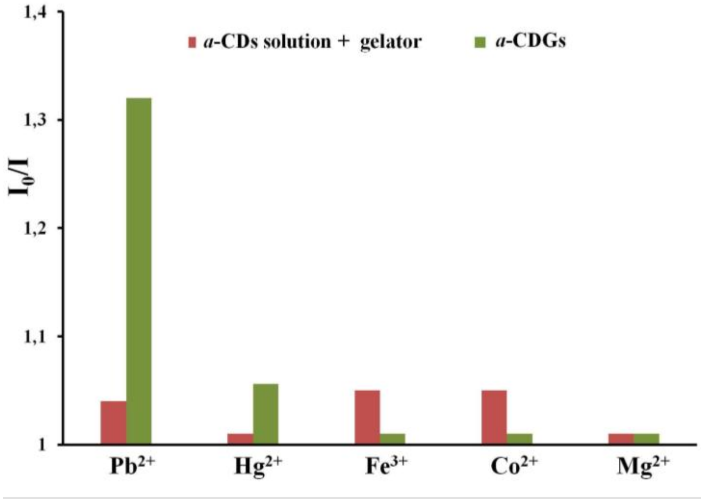

Figure S3. Photoluminescent response to 10 µg mL^{-1} of metal ions to *α*-CD solutions in the presence of dissolved gelator (1 mg mL^{-1}) and *α*-CD-containing hydrogels (*α*-CDGs) at λ_{ex} = 370 nm and λ_{em} = 445 nm.

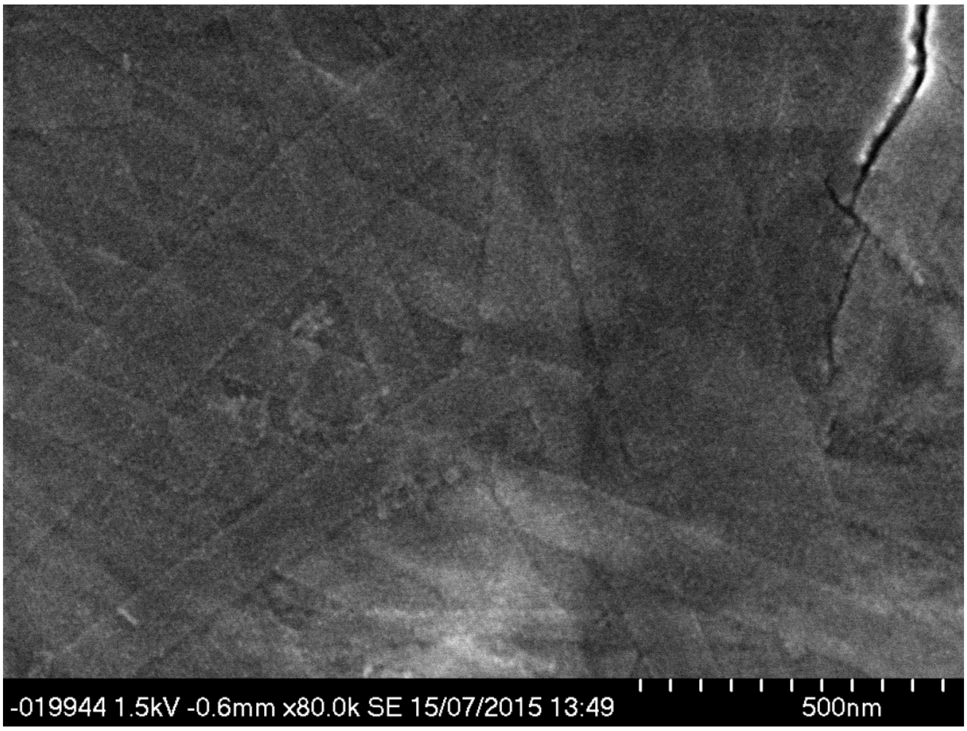

Figure S4. SEM micrograph of the chromium-coated xerogel of **1a** 1 wt%.

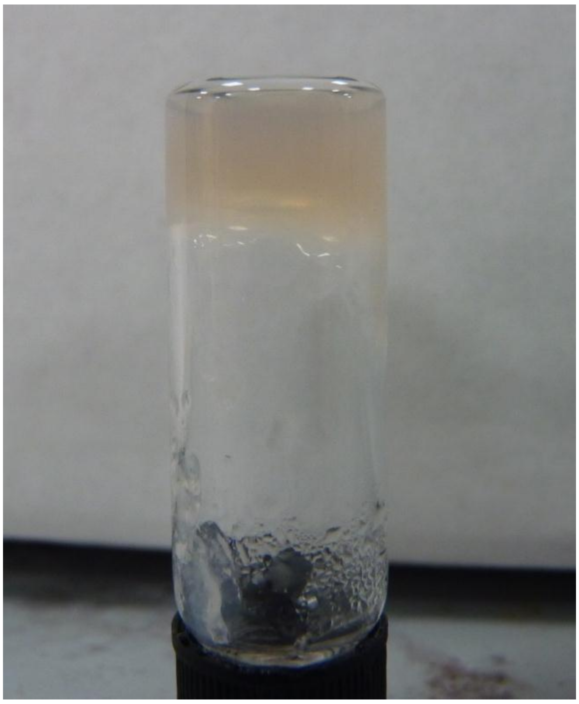

Figure S5 hydrogel of gelator **1a** at 2 wt% after standing for 1 day.

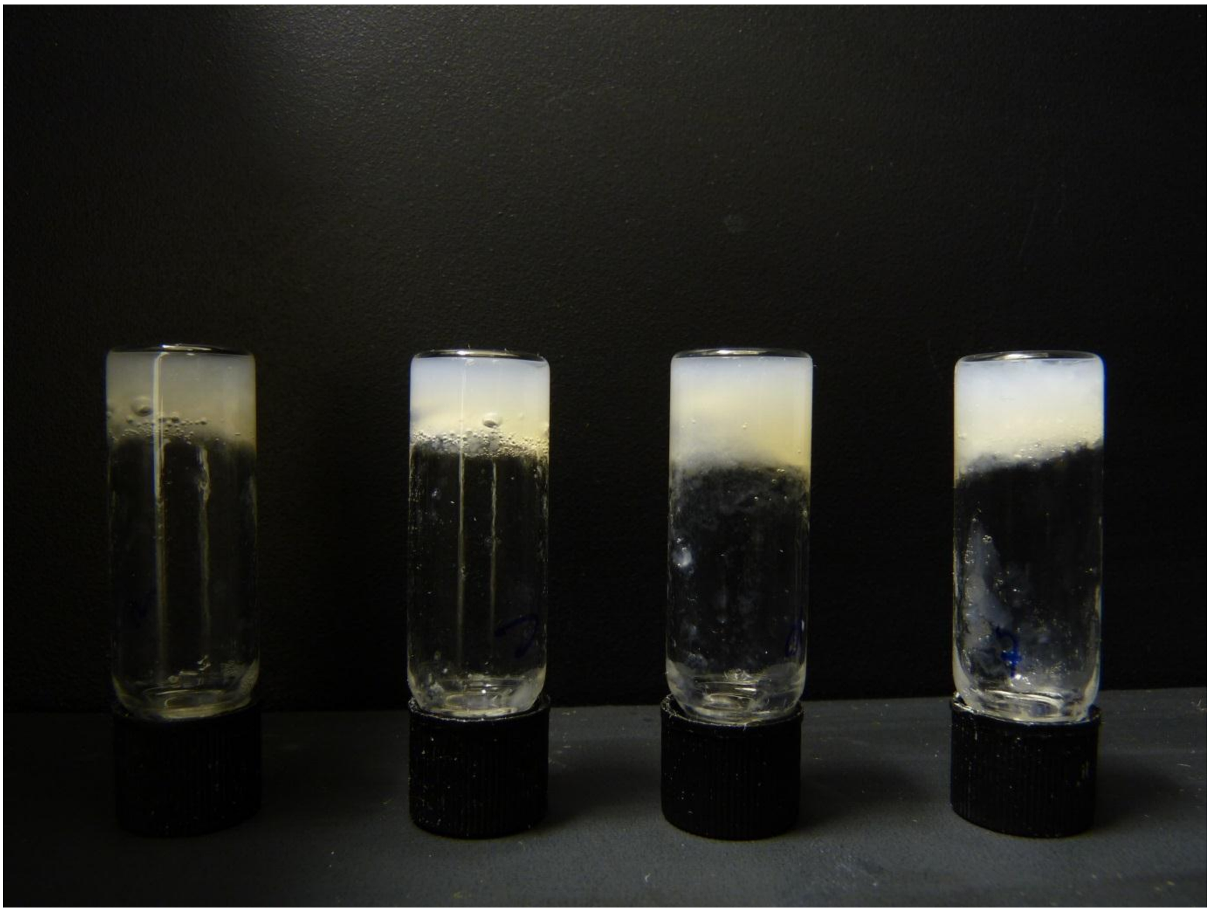

Figure S6 *a*-, *c*-, *p*- and *t*-CD gels under ambient lighting, corresponding to Figure 6a in the main paper.

Gelation and solubility studies carried out on compounds **1a**, **1b**, **2a**, and **2b** are in tables S1-S5:

Solvent	30 min	4 h	24 h	48 h	72 h
**1,2,4-trichlorobenzene**	S	S	S	S	S
**1,2-dibromoethane**	PG	PG	S	S	S
**2-butanone**	G	CG	CG	CG	CG
**1,2-dichlorobenzene**	PG	PG	PG	PG	PG
**1,3-dichlorobenzene**	S	S	S	S	S
**1,4-dioxane**	G	G	G	G	CG
**1-butanol**	S	S	G	G	G
**1-pentanol**	G	G	G	G	G
**1-propanol**	G	G	G	G	G
**2-butanol**	G	G	G	G	G
**2-Ethyl pyridine**	G	G	G	G	G
**2-Picoline**	G	G	G	G	G
**2-propanol**	G	G	G	G	G
**3-chloro-1-propanol**	G	G	G	G	G
**3-Picoline**	G	G	G	G	G
**4-Ethyl pyridine**	G	G	G	G	G
**4-Picoline**	G	G	G	G	G
**Acetic acid**	S	G	G	G	G
**Acetone**	G	CG	CG	CG	CG
**Acetonitrile**	G	CG	CG	CG	CG
**Benzene**	I	I	I	I	I
**Benzyl alcohol**	G	G	G	G	G
**Chlorobenzene**	I	I	I	I	I
**Chloroform**	P	P	P	P	P
**Cyclohexane**	I	I	I	I	I
**Cyclohexanone**	PG	PG	PG	PG	P
**Cyclopentanone**	G	G	G	G	G
**Dichloromethane**	PG	PG	G	G	G
**Diethyl ether**	I	I	I	I	I
**Diethylene glycol**	S	S	S	S	S
**Diisopropyl ether**	I	I	I	I	I
**Dimethylacetamide**	S	S	S	S	S
**DMF**	G	G	G	G	G
**DMSO**	S	S	S	S	S
**Ethanol**	G	G	G	G	G
**Ethyl acetate**	I	I	I	I	I
**Ethylene glycol**	S	S	S	S	S
**Ethylene glycol butyl ether**	G	G	G	CG	CG
**Hexane**	I	I	I	I	I
**Mesitylene**	I	I	I	I	I
**Methanol**	P	P	P	P	P
**Nitrobenzene**	G	G	G	G	G
**Nitromethane**	S	S	G	G	CG
**o-xylene**	I	I	I	I	I
**p-xylene**	I	I	I	I	I
**Pyridine**	G	G	G	G	G
**THF**	I	I	I	I	I
**Toluene**	I	I	I	I	I
**Triethylene glycol**	S	S	S	S	S
**Water**	S	S	S	S	PG

Table S1. Gelation behaviour of **1a** as a function of time. The compound (1 w.t. %) was dissolved in the relevant solvent at elevated temperature and allowed to cool to ambient temperature on the bench top. Categories are self-supporting gel according to the inversion test (G) partial gel formation (PG), collapsed gel (CG) solution (S), rapid precipitate formed from solution (P), insoluble (I). All experiments used increased temperatures to form gels.

Solvent	30 min	4 h	24 h	48 h	72 h
**1,2,4-trichlorobenzene**	I	I	I	I	I
**1,2-dibromoethane**	I	I	I	I	I
**2-butanone**	I	I	I	I	I
**1,2-dichlorobenzene**	I	I	I	I	I
**1,3-dichlorobenzene**	I	I	I	I	I
**1,4-dioxane**	I	I	I	I	I
**1-butanol**	S	S	G	G	G
**1-pentanol**	S	S	G	G	G
**1-propanol**	S	S	S	S	PG
**2-butanol**	S	S	S	S	PG
**2-Ethyl pyridine**	G	G	G	PG	PG
**2-Picoline**	S	S	S	PG	PG
**2-propanol**	S	S	S	S	PG
**3-chloro-1-propanol**	I	I	I	I	I
**3-Picoline**	G	G	G	G	G
**4-Ethyl pyridine**	S	S	G	G	G
**4-Picoline**	S	S	S	S	PG
**Acetic acid**	PG	PG	PG	PG	CG
**Acetone**	I	I	I	I	I
**Acetonitrile**	I	I	I	I	I
**Benzene**	I	I	I	I	I
**Benzyl alcohol**	G	G	G	G	G
**Chlorobenzene**	I	I	I	I	I
**Chloroform**	I	I	I	I	I
**Cyclohexane**	I	I	I	I	I
**Cyclohexanone**	S	S	S	S	PG
**Cyclopentanone**	I	I	I	I	I
**Dichloromethane**	I	I	I	I	I
**Diethyl ether**	I	I	I	I	I
**Diethylene glycol**	G	G	G	G	G
**Diisopropyl ether**	I	I	I	I	I
**Dimethylacetamide**	S	S	S	S	S
**DMF**	S	S	S	S	S
**DMSO**	S	S	S	S	S
**Ethanol**	G	G	G	G	G
**Ethyl acetate**	I	I	I	I	I
**Ethylene glycol**	G	G	G	G	G
**Ethylene glycol butyl ether**	S	S	S	S	PG
**Hexane**	I	I	I	I	I
**Mesitylene**	I	I	I	I	I
**Methanol**	PG	PG	PG	PG	PG
**Nitrobenzene**	I	I	I	I	I
**Nitromethane**	I	I	I	I	I
**o-xylene**	I	I	I	I	I
**p-xylene**	I	I	I	I	I
**Pyridine**	G	G	G	G	G
**THF**	PG	PG	PG	G	G
**Toluene**	I	I	I	I	I
**Triethylene glycol**	G	PG	PG	PG	PG
**Water**	I	I	I	I	I

Table S2. Gelation behaviour of **1b** as a function of time. The compound (1 w.t. %) was dissolved in the relevant solvent at elevated temperature and allowed to cool to ambient temperature on the bench top. Categories are self-supporting gel according to the inversion test (G) partial gel formation (PG), collapsed gel (CG) solution (S), insoluble (I). All experiments used increased temperatures to form gels.

Solvent	30 min	4 h	24 h	48 h	72 h
**1-propanol**	G	G	G	CG	CG
**2-Ethyl pyridine**	PG	PG	PG	PG	PG
**2-Picoline**	PG	G	G	G	G
**3-chloro-1-propanol**	PG	G	G	G	G
**3-Picoline**	G	G	G	G	G
**4-Ethyl pyridine**	G	G	G	G	G
**4-Picoline**	G	G	G	G	G
**Ethanol**	G	CG	CG	CG	CG
**Acetone**	G	G	CG	CG	CG
**Methanol**	PG	PG	PG	PG	PG
**Pyridine**	G	G	G	G	G

Table S3. Gelation experiments carried out on compound **1a** recorded at various times listing whether gels formed (G), a partial gel formed (PG), a collapsed gel was observed (CG) or a solution was observed (S). All experiments were carried out using only sonication as a mechanism to induce gelation.

Solvent	30 min	4 h	24 h	48 h	72 h
**1,2,4-trichlorobenzene**	S	S	S	S	S
**1,2-dibromoethane**	S	S	S	S	S
**2-butanone**	S	S	S	S	S
**1,2-dichlorobenzene**	S	S	S	S	S
**1,3-dichlorobenzene**	S	S	S	S	S
**1,4-dioxane**	S	S	S	S	S
**1-butanol**	S	S	S	S	S
**1-pentanol**	S	S	S	S	S
**1-propanol**	S	S	S	S	X
**2-butanol**	S	S	S	S	S
**2-Ethyl pyridine**	S	S	S	S	S
**2-Picoline**	S	S	S	S	S
**2-propanol**	S	S	S	S	S
**3-chloro-1-propanol**	S	S	S	S	S
**3-Picoline**	S	S	S	S	S
**4-Ethyl pyridine**	S	S	S	S	S
**4-Picoline**	S	S	S	S	S
**Acetic acid**	S	S	S	S	S
**Acetone**	I	I	I	I	I
**Acetonitrile**	I	I	I	I	I
**Benzene**	I	I	I	I	I
**Benzyl alcohol**	S	S	S	S	S
**Chlorobenzene**	I	I	I	I	I
**Chloroform**	I	I	I	I	I
**Cyclohexane**	I	I	I	I	I
**Cyclohexanone**	S	S	S	S	S
**Cyclopentanone**	S	S	S	S	S
**Dichloromethane**	I	I	I	I	I
**Diethyl ether**	I	I	I	I	I
**Diethylene glycol**	S	S	S	S	S
**Diisopropyl ether**	I	I	I	I	I
**Dimethylacetamide**	S	S	S	S	S
**DMF**	S	S	S	S	S
**DMSO**	S	S	S	S	S
**Ethanol**	S	S	S	S	S
**Ethyl acetate**	I	I	I	I	I
**Ethylene glycol**	S	S	S	S	S
**Ethylene glycol butyl ether**	S	S	S	S	S
**Hexane**	I	I	I	I	I
**Mesitylene**	I	I	I	I	I
**Methanol**	S	S	S	S	S
**Nitrobenzene**	S	S	S	S	S
**Nitromethane**	S	S	S	S	S
**o-xylene**	I	I	I	I	I
**p-xylene**	I	I	I	I	I
**Pyridine**	S	S	S	S	S
**THF**	I	I	I	I	I
**Toluene**	I	I	I	I	I
**Triethylene glycol**	S	S	S	S	S
**Water**	S	S	S	S	S

Table S4. Solubility studies of **2a** as a function of time. The compound (1 w.t. %) was dissolved in the relevant solvent at elevated temperature and allowed to cool to ambient temperature on the bench top. Categories are solution (S), insoluble (I) crystals formed (X).

Solvent	30 min	4 h	24 h	48 h	72 h
1,2,4-trichlorobenzene	I	I	I	I	I
1,2-dibromoethane	I	I	I	I	I
2-butanone	I	I	I	I	I
1,2-dichlorobenzene	I	I	I	I	I
1,3-dichlorobenzene	I	I	I	I	I
1,4-dioxane	I	I	I	I	I
1-butanol	S	S	S	S	S
1-pentanol	S	S	S	S	S
1-propanol	S	S	S	S	S
2-butanol	S	S	S	S	S
2-Ethyl pyridine	S	S	S	S	S
2-Picoline	S	S	S	S	S
2-propanol	S	S	S	S	S
3-chloro-1-propanol	S	S	S	S	S
3-Picoline	S	S	S	S	S
4-Ethyl pyridine	S	S	S	S	S
4-Picoline	S	S	S	S	S
Acetic acid	S	S	S	S	S
Acetone	S	S	S	S	S
Acetonitrile	S	S	S	S	S
Benzene	I	I	I	I	I
Benzyl alcohol	S	S	S	S	S
Chlorobenzene	I	I	I	I	I
Chloroform	I	I	I	I	I
Cyclohexane	I	I	I	I	I
Cyclohexanone	S	S	S	S	S
Cyclopentanone	S	S	S	S	S
Dichloromethane	I	I	I	I	I
Diethyl ether	I	I	I	I	I
Diethylene glycol	S	S	S	S	S
Diisopropyl ether	I	I	I	I	I
Dimethylacetamide	S	S	S	S	S
DMF	S	S	S	S	S
DMSO	S	S	S	S	S
Ethanol	S	S	S	S	S
Ethyl acetate	I	I	I	I	I
Ethylene glycol	S	S	S	S	S
Ethylene glycol butyl ether	S	S	S	S	S
Hexane	I	I	I	I	I
Mesitylene	I	I	I	I	I
Methanol	S	S	S	S	S
Nitrobenzene	I	I	I	I	I
Nitromethane	I	I	I	I	I
o-xylene	I	I	I	I	I
p-xylene	I	I	I	I	I
Pyridine	S	S	S	S	S
THF	S	S	S	S	S
Toluene	I	I	I	I	I
Triethylene glycol	S	S	S	S	S
Water	I	I	I	I	I

Table S5. Solubility studies of **2b** as a function of time. The compound (1 w.t. %) was dissolved in the relevant solvent at elevated temperature and allowed to cool to ambient temperature on the bench top. Categories are solution (S), insoluble (I) crystals formed (X).
